# Expressive suppression in psychosis: The association with social context

**DOI:** 10.1371/journal.pone.0230102

**Published:** 2020-03-11

**Authors:** UnYoung Chavez-Baldini, Marieke Wichers, Ulrich Reininghaus, Johanna T. W. Wigman, Genetic Risk and Outcome of Psychosis Investigators

**Affiliations:** 1 Faculty of Psychology and Neuroscience, Maastricht University, Maastricht, The Netherlands; 2 Interdisciplinary Center Psychopathology and Emotion Regulation (ICPE), University of Groningen, University Medical Center Groningen (UMCG), University Center Psychiatry (UCP), Groningen, The Netherlands; 3 Department of Psychiatry and Neuropsychology, School of Mental Health and Neuroscience, Maastricht University, Maastricht, The Netherlands; 4 Centre for Epidemiology and Public Health, Health Service and Population Research Department, Institute of Psychiatry, Psychology & Neuroscience, King’s College London, London, England, United Kingdom; University of Lleida, SPAIN

## Abstract

As emotion regulation deficits have been implicated in psychotic disorders, it is imperative to investigate not only the effect of regulation strategies but also how they are used. One such strategy is expressive suppression, the inhibition of emotion-expressive behavior, which may be influenced by social context. Therefore, this study aimed to investigate whether the use of expressive suppression was associated with social context and affect in daily life and if this differed between patients with psychosis and controls. Multilevel models using experience sampling method (ESM) data of 34 patients with psychotic disorders and 53 controls from the Genetic Risk and Outcome in Psychosis (GROUP) project were conducted. Expressive suppression and social context were assessed once a day for six days and daily affect was averaged per participant per day. Social context was significantly associated with the use of expressive suppression in daily life, so that the use of expressive suppression differed when in the presence of familiar versus non-familiar company when receiving negative feedback. This finding did not differ between patients and controls. This demonstrates that taking the situation into account when studying expressive suppression, and emotion regulation in general, may improve our understanding of how regulation takes place.

## Introduction

Individuals with psychosis experience deficits in adaptive emotion regulation, which may be related to psychotic symptoms and other difficulties [1]. Furthermore, maladaptive emotion regulation strategies have been shown to mediate the path between adverse life events and positive psychotic symptoms, such as delusion proneness and hallucinations [2] and to sometimes increase the severity of hallucinations [3]. Therefore, it is important to elucidate the nature of emotion regulation in psychosis, including how patients with psychotic disorders employ emotion regulatory strategies.

Broadly, emotion regulation refers to goal-directed behaviors meant to influence various aspects of emotion generation [4, 5]. These behaviors can affect which emotions individuals experience, when and how they are experienced, and how they are expressed [6]. Specifically, response modulation is a type of emotion regulation strategy that focuses on influencing the emotional response after the emotion is developed [4]. One such response modulation is expressive suppression, which focuses on inhibiting or reducing emotion-expressive behavior when emotionally aroused [7]. While expressive suppression is effective in decreasing the behavioral expression of negative emotions, it has been shown to be ineffective in the regulation of negative emotions as it tends to increase negative affect and decrease positive affect [7–9]. Thus, the overuse of expressive suppression may be maladaptive as is supported by findings that habitual expressive suppression is associated with worse well-being [7, 10].

There is mixed evidence on the possible overreliance on expressive suppression in those who have psychotic disorders. Some research suggests that they are more likely to use expressive suppression in comparison to healthy controls [11, 12] whereas other research finds no differences [13, 14]. However, many previous studies assess expressive suppression as a habitual concept, but the use of expressive suppression may vary greatly within-person according to the context [9]. Therefore, it is necessary to also investigate expressive suppression as a variable strategy to elucidate how individuals with psychosis employ expressive suppression.

Emotion regulation is not solely an individual process but may be influenced by social context and interpersonal interactions [15, 16]. In interpersonal interactions, individuals may rely on a safety signal from the other person to properly share their emotions and appraise the event that caused their emotions, meaning that the emotion regulation is response-dependent as it relies on another person’s feedback [15]. This may also apply to expressive suppression. There is, however, a dearth of research on how social context can influence the use of expressive suppression. Research that has investigated the influence of social context on the use of expressive suppression has found that individuals tend to use more suppression when they are lower in social hierarchy [17]. In this case, expressive suppression could protect against negative outcomes of showing expression in “unsafe” situations, such as when one is low in the social hierarchy. However, it could be that if one suppresses their emotional expression in “safe” situations, one feels a sense of inauthenticity that may lead to negative consequences [7]. Furthermore, a recent study demonstrated in a sample of college students that expressive suppression can indeed be influenced by company in that less expressive suppression is used in familiar company [18].

However, the influence of social context on the use of expressive suppression and the influence of context on the emotional consequences of expressive suppression have yet to be explored in patients with psychosis. Patients with psychosis tend to have social cognitive deficits, such as with facial affect recognition [19], which may impact how they perceive their social environment. Furthermore, patients tend to have a higher negative reactivity to daily life stressors [20], and so expressive suppression may have a more marked effect on their affect as compared to controls.

Therefore, the present study aimed to investigate expressive suppression as a response-dependent strategy in the context of negative events; specifically, whether the use of expressive suppression is influenced by social context and how this impacts on affect for patients with psychosis and controls in daily life. In doing so, expressive suppression was assessed as a dynamic strategy while acknowledging the interpersonal aspect of emotion regulation. This could perhaps provide some insight into how individuals with psychotic disorders use expressive suppression, allowing for interventions that target this.

The study investigated two questions: 1) the association between two aspects of social context (company and feedback) and the use of expressive suppression and whether this differs between patients and controls, and 2) the association between expressive suppression and daily affect according to social context (company and feedback) and whether this differs between patients and controls.

It was expected that familiar company and/or positive feedback would be associated with lower expressive suppression. Furthermore, this was expected to differ between patients and controls, such that the association between social context and expressive suppression will be greater in controls than in patients. It was also hypothesized that higher expressive suppression with familiar company and/or positive feedback (e.g., “safe” situations) would be related to higher negative affect and lower positive affect. It was expected that this would also differ between patients and controls in that the association between expressive suppression and affect will be greater in patients than in controls.

## Methods

### Participants

The sample of this study consisted of 34 patients with psychotic disorders and 53 controls from wave 3 (six-year follow-up) of the Genetic Risk and Outcome in Psychosis (GROUP) project [21]. Data pertain to follow-up measures of the ongoing longitudinal study (GROUP) in Europe. In selected representative geographical areas in the Netherlands and Belgium, patients were identified through clinicians whose caseload was screened for inclusion criteria. Subsequently, a group of patients presenting consecutively at these services either as out-patients or in-patients were recruited for the study. Controls were selected through a system of random mailings to addresses in the catchment areas of the cases. The full GROUP sample consisted of 3684 participants (patients, relatives, and controls) between the ages of 16–50 years, excluding parents. Patients had a diagnosis of a schizophrenia or psychotic disorder as per the Diagnostic and Statistical Manual of Mental Disorders, Fourth Edition (DSM-IV) criteria [22], and the healthy controls had to have no lifetime psychotic disorder as well as no first- or second-degree relative with a psychotic disorder [21]. All participants had to be able and willing to provide written informed consent, which was obtained from all participants before the start of the first assessment [21].

The study protocol was approved centrally by the Ethical Review Board of the University Medical Center Utrecht (#NL46405.018.13) and subsequently by local review boards of each participating institute: Academic Psychiatric Centre of the Academic Medical Center and the mental health institutions: GGZ Ingeest, Arkin, Dijk en Duin, GGZ Rivierduinen, Erasmus Medical Centre, GGZ Noord Holland Noord; University Medical Center Groningen and the mental health institutions: Lentis, GGZ Friesland, GGZ Drenthe, Dimence, Mediant, GGNet Warnsveld, Yulius Dordrecht and Parnassia psycho-medical center The Hague; Maastricht University Medical Centre and the mental health institutions: GGzE, GGZ Breburg, GGZ Oost-Brabant, Vincent van Gogh voor Geestelijke Gezondheid, Mondriaan, Virenze riagg, Zuyderland GGZ, MET ggz, Universitair Centrum Sint-Jozef Kortenberg, CAPRI University of Antwerp, PC Ziekeren Sint-Truiden, PZ Sancta Maria Sint-Truiden, GGZ Overpelt, OPZ Rekem; University Medical Center Utrecht and the mental health institutions Altrecht, GGZ Centraal and Delta. All participants in this study gave written informed consent after full verbal and written information about the study, and each participant is identified with a unique research identification to maintain confidentiality of data.

This study focused on a sample of GROUP that participated in an experience sampling method study (ESM), which had a full sample of 465 participants (patients, relatives, and controls). Participants were included in the final sample if they had sufficient ESM data on expressive suppression and social feedback (i.e., completed a minimum of five evening questionnaires and reported having a minimum of three conversations; n = 166). Furthermore, only the observations of participants who were in either the control or patient group were included, leading to the final sample (n = 87).

### Measures

#### Demographics and clinical information

Demographic and clinical information regarding gender, age, ethnicity, and group (i.e., control versus patient) for the participants was acquired from the GROUP database, release number 1.0.

The following items were from the ESM protocol that the patients completed wave 3 of the GROUP study. ESM is a structured diary method with high ecological validity that uses both self-report scales and open-ended questions on the participant’s feelings, thoughts, and symptoms as well as the contexts in which these occur [23]. This method allows for the capturing of moment-to-moment and daily patterns within individuals, giving room for heterogeneity of patterns whilst also enabling the analysis of patterns at the group level. Participants are randomly prompted by a beep signal to complete the ESM questionnaire ten times a day and a separate evening questionnaire once a day for six days. The evening questionnaire asked participants questions regarding a negative and a positive event that occurred during the day. The items of the evening questionnaire about to the negative event were used.

#### Affect

Participants rated adjectives about affect on a 7-point Likert scale (1 = not at all to 7 = very much) ten times a day. The positive affect scale consists of four adjectives (cheerful, relaxed, satisfied, and enthusiastic), and the negative affect scale consists of five adjectives (insecure, down, lonely, anxious, annoyed).

#### Expressive suppression

Expressive suppression was measured in the evening questionnaire (i.e., once a day) with one item “I showed my emotions” that was rated on a 7-point Likert scale (1 = not at all to 7 = very much). This item was prompted as a coping strategy in relation to the negative event indicated by the participant. A lower rating denotes more expressive suppression.

#### Social context (company and feedback)

Social context was assessed with two different items from the evening questionnaire. The first item asked the participant about their company (“With whom was this?” with “this” referring to the most negative event that occurred that day), and the answers were categorized into two types: with familiar individuals (family members, friends, partner) and with non-familiar individuals work colleagues, acquaintances, strangers, other; in accordance with Verdoux, Husky [24]. The category of “alone” was not included in analyses as this study focused on social interactions. The second item assessed feedback (“This person responded with understanding”) on a 7-point Likert scale (1 = not at all to 7 = very much), which was in relation to a conversation about the negative event.

### Procedure

This study analyzed the ESM data from a sample of the GROUP wave 3 (six-year follow-up) database containing results from participants across different sites. Except for the diagnostic instrument, the same measures were used across all the sites and the data was gathered into a centralized coded database [21]. The assessments were administered by trained research assistants and took place at a mental health center or academic center. Permission to use the data was first acquired from the GROUP coordinators before proceeding with data analyses. Once that was completed, analyses of the data were conducted with the Stata 13.1 software program.

To associate the daily repeated measurements of affect with the single measures of the evening questionnaire items, such as expressive suppression, the scores of the positive and negative affect items were aggregated separately per day per participant. This was done because the evening questionnaire itself did not include questions on affect; therefore, a daily positive affect sum score and a daily negative affect sum score were used in relation to the evening questionnaire.

### Statistical analysis

To test the hypotheses, multilevel analyses were conducted, which are suitable for the hierarchical nature of ESM data in which the observations (level-1) are nested within individuals (level-2; [25]). For all statistical tests, an alpha level of 0.05 was used.

Before testing the hypotheses, basic descriptive statistics were conducted to determine the sample characteristics. Gender, age, and ethnicity were considered a priori as potential confounders. They were analyzed with linear regression or chi-square tests as appropriate to test for group differences to determine if they were significantly different and, therefore, needed to be included as a confounder. The main ESM variables (e.g., expressive suppression) were aggregated per participant and tested for group differences with regression, independent sample t-test for unequal variances, or chi-square tests.

The multilevel model of the association of social context on expressive suppression was analyzed with company as a categorical predictor, feedback as a continuous predictor, and expressive suppression as the outcome. Using a likelihood ratio test, this model was tested against a model with the interaction company x feedback as a predictor and expressive suppression as the outcome. Group was added as a level-2 moderator in a subsequent analysis, and the main effects model was tested against the interaction effect model. Interactions were probed by investigating the simple slopes of marginal effects.

The association between expressive suppression and affect depending on social context was analyzed with separate multilevel models with the aggregated daily positive affect and the aggregated daily negative affect as the outcomes. The predictors were interaction terms for social context and expressive suppression (i.e., company x expressive suppression, feedback x expressive suppression, company x feedback x expressive suppression). The interactions models were tested against the main effects models using likelihood ratio tests. To avoid complex interactions, associations were stratified between patients and controls to investigate these relationships in the two groups.

## Results

### Sample characteristics

Table 1 shows the sample characteristics. Participants in the control group were older than those in the patient group, and there were more women than men in each group and in the sample overall. The groups did not exhibit a significant difference in ethnicity, however, and the sample consisted mostly of Caucasian participants. Therefore, age and gender were included as confounders in all the multilevel models. It should also be noted that 27 out of 34 patients were in remission. Furthermore, there was no significant difference in the use of expressive suppression between patients and controls. The groups also did not vary in in terms of company nor feedback. However, patients reported experiencing less positive affect and more negative affect compared to controls. Lastly, remitted patients and non-remitted patients did not have any significant differences regarding the main outcome measures and there were no differences in any of the main outcome variables between diagnoses in the patient group.

**Table 1:** Sample characteristics.

	Patients (n = 34)	Controls (n = 53)	Test statistic	p
Age (years), mean (SD)	33.9 (7.5)	39.8 (12.1)	F = 6.39, df = 1	.013
Gender, n (%)			χ 2 = 10.48, df = 1	.001
Male	15 (44.1)	7 (13.2)		
Female	19 (55.8)	46 (86.8)		
Ethnicity, Caucasian (%)	32 (94.1)	52 (98.1)	χ 2 = 0.99, df = 1	.319
Diagnosis, n (%)				
SZ, paranoid type	12 (35.3)			
SZ, undifferentiated type	2 (5.9)			
Psychosis with delusions	1 (2.9)			
Psychosis NOS	5 (14.7)			
Schizoaffective disorder	14 (41.2)			
In remission, n (%)	27 (79.4)			
Expressive suppression, mean (SD)	3.9 (1.2)	3.8 (1.3)	F = 2.03, df = 1	.155
Type of diagnosis^a^			F = 2.02, df = 4	.118
Remission status (yes/no)^a^			t = -0.32, df = 8.39	.754
Company, n (%)			χ 2 = 0.10, df = 1	.749
Familiar	82 (59.9)	155 (61.5)		
Non-familiar	55 (40.1)	97 (38.5)		
Type of diagnosis^a^			χ 2 = 5.44, df = 4	.245
Remission status (yes/no)^a^			χ 2 = 0.62, df = 1	.432
Feedback, mean (SD)	5.3 (0.9)	5.4 (0.7)	F = 0.56, df = 1	.453
Daily PA, mean (SD)	126.8 (41.4)	148.7 (43.0)	F = 23.52, df = 1	<.001
Type of diagnosis^a^			F = 1.83, df = 4	.149
Remission status (yes/no)^a^			t = -1.52, df = 12.54	.154
Daily NA, mean (SD)	59.4 (21.2)	54.0 (21.0)	F = 5.81, df = 1	.016
Type of diagnosis^a^			F = 1.25, df = 4	.314
Remission status (yes/no)^a^			t = -0.15, df = 7.04	.887

SD, standard deviation; df, degrees of freedom

^a^ Statistical difference between diagnoses and state of remission analyzed only in patients

### Association between social context and the use of expressive suppression

The interaction between company and feedback was significantly associated with the use of expressive suppression and exhibited better model fit according to the likelihood ratio test. Estimation of the simple slopes of marginal effects demonstrated that when receiving positive feedback, company did not matter for expressive suppression (B = 0.21, 95% CI [-0.45, 0.88], *p* = .527), but when receiving negative feedback, participants used less expressive suppression in familiar company as compared to non-familiar company (B = -2.02, 95% CI [-3.43, -0.62], *p* = .005). Furthermore, there was no effect of group on the association between social context (company and feedback) and expressive suppression. These results are demonstrated in Table 2.

**Table 2:** The Association between social context and the use of expressive suppression^a^.

Outcome: Expressive Suppression				
Social Context	adj. β (95% CI)	p	LR test^b^^,^^c^	
			χ2 (df)	p
Company	-2.40 (-4.11–-0.69)	.006		
Feedback	-0.16 (-0.35–0.03)	.103		
Company x Feedback	0.37 (0.06–0.69)	.020	5.34 (1)	.021
Company x Group	-1.04 (-4.6–2.52)	.567		
Feedback x Group	-0.05 (-0.45–0.35)	.797		
Company x Feedback x Group	0.16 (-0.49–0.81)	.633	6.15 (4)	.188

CI, confidence interval; df, degrees of freedom; LR, likelihood ratio

^a^ Adjusted for age and gender

^b^ LR test (a nested in b): model a (company, feedback) vs model b (company x feedback)

^c^ LR test (a nested in b): model a (company, feedback, group) vs model b (company x feedback x group)

### Association of expressive suppression and affect depending on social context

The results indicated that there was no significant interaction effect of expressive suppression with company and/or feedback on positive affect (Tables 3 and 4). These effects were non-significant for patients and for controls.

**Table 3:** The association of expressive suppression and positive affect depending on social context^a^.

Outcome: Daily positive affect				
Social Context	adj. β (95% CI)	p	LR test^b^^,^^c^^,^^d^	
			χ2 (df)	p
Company x Expressive suppression	3.208 (-1.74–8.15)	.204	1.61 (1)	.204
Feedback x Expressive suppression	0.855 (-1.27–2.97)	.430	0.62 (1)	.431
Company x Feedback x Expressive suppression	0.010 (-4.80–4.82)	.997	5.02 (4)	.285

CI, confidence interval; df, degrees of freedom; LR, likelihood ratio

^a^ Adjusted for age and gender

^b^ LR test (a nested in b): model a (company, expressive suppression) vs model b (company x expressive suppression)

^c^ LR test (a nested in b): model a (feedback, expressive suppression) vs model b (feedback x expressive suppression);

^d^ LR test (a nested in b): model a (company, feedback, expressive suppression) vs model b (company x feedback x expressive suppression)

**Table 4:** Stratified associations between groups (Table 3).

Social Context	Patients		Controls	
	adj. β (95% CI)	p	adj. β (95% CI)	p
Company x Expressive suppression	-19.57 (-55.02–15.88)	.279	3.62 (-32.77–40.01)	.845
Feedback x Expressive suppression	-1.92 (-5.9–2.06)	.345	3.00 (-0.37–6.37)	.081
Company x Feedback x Expressive suppression	4.77 (-1.90–11.44)	.161	-0.40 (-6.92–6.13)	.905

There was also no significant interaction effect of expressive suppression with company and/or feedback on negative affect (Tables 5 and 6). These effects were non-significant for patients and for controls.

**Table 5:** The association of expressive suppression and negative affect depending on social context^a^.

Outcome: Daily negative affect				
Social Context	adj. β (95% CI)	p	LR test^b^^,^^c^^,^^d^	
			χ2 (df)	p
Company x Expressive suppression	-0.021 (-2.76–2.71)	.988	0.00 (1)	.988
Feedback x Expressive suppression	0.050 (-1.06–1.16)	.929	0.01 (1)	.929
Company x Feedback x Expressive suppression	-0.391 (-2.89–2.11)	.759	0.53 (4)	.970

CI, confidence interval; df, degrees of freedom; LR, likelihood ratio

^a^ Adjusted for age and gender

^b^ LR test (a nested in b): model a (company, expressive suppression) vs model b (company x expressive suppression)

^c^ LR test (a nested in b): model a (feedback, expressive suppression) vs model b (feedback x expressive suppression);

^d^ LR test (a nested in b): model a (company, social feedback, expressive suppression) vs model b (company x feedback x expressive suppression)

**Table 6:** Stratified associations between groups (Table 5).

Social Context	Patients		Controls	
	adj. β (95% CI)	p	adj. β (95% CI)	p
Company x Expressive suppression	-11.63 (-33.79–10.52)	.303	8.40 (-8.35–25.15)	.326
Feedback x Expressive suppression	-1.17 (-3.66–1.31)	.356	0.90 (-0.65–2.44)	.256
Company x Feedback x Expressive suppression	1.53 (-2.64–5.70)	.472	-1.24 (-4.24–1.76)	.418

## Discussion

In this study, expressive suppression was investigated in daily life as a response-dependent interpersonal emotion regulation strategy. This was done by analyzing the association between the daily measurement of expressive suppression to positive affect, negative affect, and social context (company and feedback) associated with a negative event on which participants reported.

Supporting the hypothesis of expressive suppression being associated with social context, an interesting interaction between company and feedback was demonstrated. Company mattered for negative feedback in that less expressive suppression was used in familiar company as compared to non-familiar company. However, company did not matter for expressive suppression when there was positive feedback. Therefore, a possible interpretation of these findings is that participants feel comfortable enough to express their emotions when with familiar company, regardless of how the other person responds, but when with non-familiar company, participants only feel comfortable expressing their emotions when they receive positive feedback.

These findings point to the social nature of expressive suppression. It can be argued that in comparison to other emotion regulation techniques, such as cognitive reappraisal and distraction, expressive suppression may rely more on social context as was also found in the English, Lee [18] study. Because individuals with psychosis tend to have impairments in facial affect recognition [19], which could have impacted how patients perceived the feedback received, we thought that patients and controls might differ in their sensitivity to the social context. However, the results indicate that there was no significant difference between patients and controls.

Finally, there was no association between expressive suppression and either positive or negative affect according to social context. This may be due to methodology rather than a reflection of the impact of expressive suppression as affect was not assessed in direct relation to the negative event. Otherwise, it may be that social context does not influence the impact of expressive suppression on either positive or negative affect. If it is the latter case, then the internal consequences of expressive suppression (i.e., affect) may not be influenced by social context. Therefore, it may be important to make a distinction between external and internal influences and consequences when it comes to expressive suppression.

The findings for both hypotheses did not differ between patients and controls, suggesting that social context influences the use of expressive suppression in similar ways for both patients and controls. This lack of differences could be explained by the fact that the ESM data was collected at six-year follow-up and most of the patients included in this study were then in remission. Thus, it is possible that remitted patients are comparable to controls in the use of expressive suppression. This could imply that expressive suppression is subject to change and that there might be possible improvements in this area. For instance, it has been shown that the frequency of expressive suppression decreases with treatment, albeit for social anxiety [26]. There were also no significant differences in the main outcome measures between remitted and non-remitted patients, which could suggest that the patient group is relatively well-functioning. This may be an effect of choosing participants who completed enough questionnaires or these measures are not very sensitive to illness severity. However, these explanations are tentative, and more research is needed to further investigate these speculations.

The results of this study should be considered preliminary and interpreted with some methodological considerations in mind. First, many participants and observations had to be excluded to obtain a valid sample for the questions of this study, leading to a relatively small sample. Especially for models including interaction terms, the analyses may have been underpowered as the numbers of participants in the individual cells were quite small. Second, expressive suppression and social context were measured once at the end of the day, which may not directly capture the dynamics of an individual in a given context but rather reflect on the whole day: the evaluation of the day could then be influenced by other events during the day and potentially increase or decrease the impact of the chosen negative event. It is therefore unknown how the negative event was specifically related to the level of positive and negative affect, which may also explain some of the null findings. Third, this study investigated responses only to negative events although it has been suggested to investigate responses to positive events as well [18]. However, negative events are focused on given its greater clinical relevance as the reactivity to daily stressors plays an important role in psychosis. For instance, it is fairly established that there is an affective pathway from daily stressors to psychotic symptoms [27, 28]; the affective reaction to a stressor may then be influenced by emotion regulation. Nonetheless, positive events should be studied as this knowledge could contribute to the understanding of expressive suppression and the regulation of emotions in psychosis, which may be of scientific interest.

This study adds to the understanding of expressive suppression as an emotion regulation strategy through a daily life perspective, measuring it as a variable strategy rather than as habitual strategy. This was done with ESM, which is more ecologically valid as opposed to other methods [29,30], such as investigating experimentally-induced expressive suppression in lab settings or measuring expressive suppression with a one-time questionnaire, such as the Emotion Regulation Questionnaire (ERQ; 7). Furthermore, while this study investigates only two aspects of social context, including social context acknowledges that emotion regulation does not occur in a vacuum and that one’s surroundings, including other people, can influence how expressive suppression is used by individuals. In addition, investigating these questions in a sample with patients is imperative as deficits in emotion regulation are apparent in psychosis. This study extends previous work in emotion regulation, specifically expressive suppression, using ESM (e.g., 9, 18) by including a clinical sample.

Further research can first focus on replicating our results with a larger sample and more measurement periods is necessary for more concrete evidence. Second, future studies should investigate the use of expressive suppression and other emotion regulation techniques over the course of illness in a clinical population with psychotic disorders to understand if and how they change over time. Third, considering the mechanisms of emotion regulation on affective disturbances in different contexts and how they lead to specific psychotic symptoms could give a more nuanced perspective of the development and maintenance of psychotic psychopathology. Overall, these future studies could lead to more fully-fledged models by taking the different stages, pathways, and processes that play a role in psychosis.
